# 3′-End Sequencing for Expression Quantification (3SEQ) from Archival Tumor Samples

**DOI:** 10.1371/journal.pone.0008768

**Published:** 2010-01-19

**Authors:** Andrew H. Beck, Ziming Weng, Daniela M. Witten, Shirley Zhu, Joseph W. Foley, Phil Lacroute, Cheryl L. Smith, Robert Tibshirani, Matt van de Rijn, Arend Sidow, Robert B. West

**Affiliations:** 1 Department of Pathology, Stanford University Medical Center, Stanford, California, United States of America; 2 Department of Genetics, Stanford University Medical Center, Stanford, California, United States of America; 3 Department of Statistics, Stanford University, Stanford, California, United States of America; 4 Department of Health Research and Policy, Stanford University Medical Center, Stanford, California, United States of America; 5 Pathology and Laboratory Service, Palo Alto Veterans Affairs Health Care System, Palo Alto, California, United States of America; The University of Hong Kong, Hong Kong

## Abstract

Gene expression microarrays are the most widely used technique for genome-wide expression profiling. However, microarrays do not perform well on formalin fixed paraffin embedded tissue (FFPET). Consequently, microarrays cannot be effectively utilized to perform gene expression profiling on the vast majority of archival tumor samples. To address this limitation of gene expression microarrays, we designed a novel procedure (3′-end sequencing for expression quantification (3SEQ)) for gene expression profiling from FFPET using next-generation sequencing. We performed gene expression profiling by 3SEQ and microarray on both frozen tissue and FFPET from two soft tissue tumors (desmoid type fibromatosis (DTF) and solitary fibrous tumor (SFT)) (total n = 23 samples, which were each profiled by at least one of the four platform-tissue preparation combinations). Analysis of 3SEQ data revealed many genes differentially expressed between the tumor types (FDR<0.01) on both the frozen tissue (∼9.6K genes) and FFPET (∼8.1K genes). Analysis of microarray data from frozen tissue revealed fewer differentially expressed genes (∼4.64K), and analysis of microarray data on FFPET revealed very few (69) differentially expressed genes. Functional gene set analysis of 3SEQ data from both frozen tissue and FFPET identified biological pathways known to be important in DTF and SFT pathogenesis and suggested several additional candidate oncogenic pathways in these tumors. These findings demonstrate that 3SEQ is an effective technique for gene expression profiling from archival tumor samples and may facilitate significant advances in translational cancer research.

## Introduction

The development of gene expression microarrays in the mid-1990s represented a significant technical achievement that, for the first time, permitted the systematic genome-wide evaluation of gene expression [1], [2]. Since their introduction, these technologies have been widely used for gene expression profiling of cancer samples, leading to the identification of gene expression patterns that predict the biological and clinical features of a wide range of human malignancies [3]–[16].

Despite the large numbers of gene expression profiling experiments performed on human cancers, the full potential of these technologies for impacting the clinical management of cancer patients has not yet been realized [17]–[21]. A major limitation of gene expression microarrays for translational cancer research is that they rely on the availability of fresh frozen tissue and show inconsistent performance on formalin fixed paraffin embedded tissue (FFPET) [22]–[27]. Consequently, gene microarrays cannot be used effectively on the vast majority of tumor specimens, since few samples are stored frozen. In contrast, essentially all tumor samples are stored as FFPET in pathology laboratories around the world [28]. In an attempt to utilize this rich source of human tumor samples, investigators have resorted to measuring the expression of relatively small numbers of known transcripts from FFPET through the use of a variety of targeted approaches, including reverse transcriptase-polymerase chain reaction (RT-PCR) [29], [30] and cDNA-mediated annealing, selection, extension and ligation (DASL) [31], [32]. No technique currently exists for accurate quantitative genome-wide expression profiling from FFPET.

In the past several years, there have been major advances in sequencing technologies, resulting in the development of ultra high-throughput sequencing (UHTS) platforms that have allowed significant increases in sequencing throughput and decreases in sequencing cost [33], [34]. There is considerable hope in the scientific community that UHTS will overcome the major limitations of microarray technology and revolutionize the field of functional genomics [35], [36]. UHTS has been developed in several platforms, including Roche 454, Illumina Genome Analyzer, and ABI SOLID. These technologies have been used to sequence human genomes [37], [38], study the genome wide binding of transcription factors [39], [40] and nucleosomes [41], characterize genome methylation patterns [42], and have been recently applied to the sequencing of transcriptomes (RNA-Seq) [35], [36], [43]–[45].

Standard RNA-Seq protocols target the entire gene transcript by either synthesizing the full-length cDNA using oligo-dT reverse primers followed by fragmentation of cDNA, or by selecting poly-A-tailed mRNA followed by RNA fragmentation and cDNA synthesis using random hexamer oligonucleotides. Since these techniques attempt to sequence the entire RNA transcript, the successful application of these methods for expression profiling depends on the presence of high quality starting total RNA.

No technique currently exists for accurate quantitative genome wide expression profiling from FFPET in which RNA has been extensively degraded. We describe here the 3SEQ assay for precise quantification of genome-wide expression levels on both frozen tissue and FFPET. In this report, we perform gene expression profiling on a collection of frozen and FFPET samples from two soft tissue tumor types (desmoid type fibromatosis (DTF) and solitary fibrous tumor (SFT)) using both Human Exonic Evidence Based Oligonucleotide (HEEBO) microarrays (http://microarray.org/sfgf/heebo.do) and 3SEQ. We assess the performance of these two gene expression profiling modalities for making reliable gene expression measurements and for identifying differentially expressed genes and biological pathways from both frozen tissue and FFPET.

## Results

### DTF and SFT Tumor Samples Selected for Gene Expression Profiling

DTF and SFT are two subtypes of fibroblastic soft tissue tumors, which show morphologic similarities, but demonstrate distinct clinical features [46]. The gene expression patterns of DTF and SFT have been previously studied in our laboratory by microarray [16], [47], [48], and these studies have revealed that although DTF and SFT are both fibroblastic tumors with similar morphologic features, they show distinct gene expression patterns. These tumors represent excellent sources of RNA for evaluating a new gene expression profiling modality, since in contrast to most carcinomas, DTF and SFT are both composed of a relatively homogenous population of tumor cells with few contaminating non-neoplastic cells, resulting in the production of distinct gene expression patterns.

The current study included a total of 23 samples, which were each profiled using at least 1 of the 4 platform-tissue type combinations (3SEQ-frozen, 3SEQ-FFPET, HEEBO-frozen, HEEBO-FFPET): the HEEBO-frozen analysis included 17 samples (DTF n = 9, SFT n = 8); the HEEBO-FFPET included 14 samples (DTF n = 6, SFT n = 8); 3SEQ-frozen included 11 samples (DTF n = 5,SFT n = 6); and 3SEQ-FFPET included 14 samples (DTF n = 6, SFT = 8) (Table S1).

### 3SEQ Sample Preparation and Sequencing

**Figure 1:**
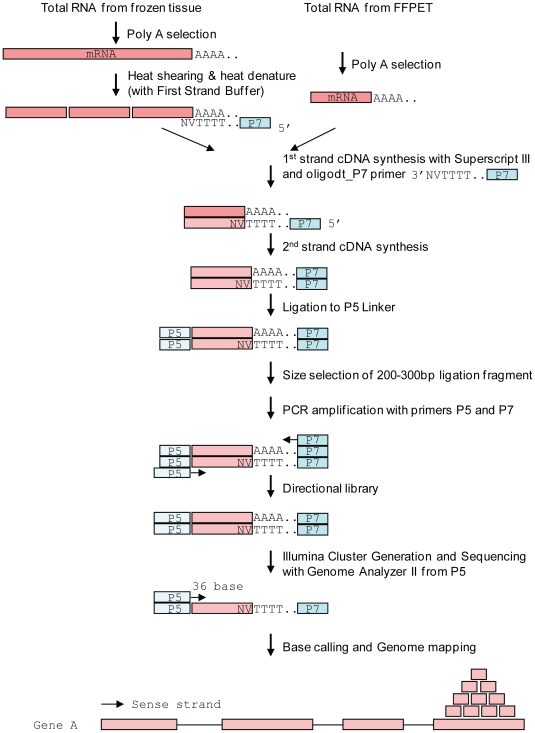
3′-end Sequencing for Expression Quantification (3SEQ) schematic. Either intact mRNA from frozen tissue or degraded mRNA from FFPET is enriched by poly-A selection. The mRNA from frozen tissue is then heat fragmented to approximately 100–200 bases. This heat fragmentation is incorporated with the RNA heat denature in the 1^st^ strand cDNA synthesis by including the 1^st^ strand cDNA buffer which contains Mg that is required for fragmentation. The short mRNA from FFPET is converted directly to cDNA without fragmentation. The 1^st^ strand cDNA is synthesized with an oligo-dT_P7 RT primer that consists of three parts: 25-oligo-dT, P7 sequence linked to oligo-dT at the 5′ end and two degenerate nucleotides NV at the 3′ end. The single stranded cDNA is then converted to double stranded cDNA and the P5 linker is ligated to the end of the cDNA fragment opposite the P7 linker. The linker-ligated cDNA fragments of approximately 250 bp are selected and a PCR reaction is performed with primers that hybridize to the P5 and P7 linkers. The sequencing library is unidirectional and composed of cDNA, the P7 linker adjacent to the poly-A tail and the P5 linker on the opposite end of the fragment. The library is sequenced from the P5 end to generate 36 bp reads by a synthesis procedure using the Illumina Genome Analyzer. The first 25 bp of each read is used to map the reads to the genome. These reads are expected to be mapped towards to the 3′ UTR or the 3′ end of the 3′-most exon of expressed genes.

The 3SEQ method is a novel type of RNA-Seq designed for accurate and quantitative genome wide expression profiling from both high quality and degraded total RNA by targeting the 3′ end of mRNA. A schematic illustration of the 3SEQ assay is shown in Figure 1. mRNA is first enriched from total RNA by poly-A selection to remove the ribosomal RNA and other non-poly-A RNA. The mRNA from fresh frozen tissue (which is intact and long) is then fragmented to 100–200 bases. The heat fragmentation of mRNA is incorporated and combined with the denature step of 1^st^ strand cDNA synthesis in the presence of Mg contained in the 1^st^ strand cDNA buffer. In contrast, the short mRNA from FFPET is converted to cDNA directly without any further fragmentation. The oligo-dt_P7 RT primer used for 1^st^ strand cDNA synthesis contains the 25-T P7 sequence at the 5′ end. The double-stranded cDNA is then ligated to the P5 adapter at the 5′ end, size selected, and amplified by PCR using primers to P5 and P7. The resulting directional library is then sequenced from the P5 end using the Illumina Genome Analyzer II.

The primary difference between the 3SEQ protocol described here and standard RNA-Seq is that standard RNA-Seq attempts to generate a sequencing library that spans the entire transcript length. Standard RNA-Seq has been applied to mutation identification and transcriptional profiling. While sequencing of the entire transcript provides abundant biological information, a significant limitation of this technique is it requires high quality starting RNA. In contrast, the aim of 3SEQ is to perform genome-wide expression quantification, which can be performed on both high quality starting RNA as well as degraded RNA. While standard RNA-Seq targets the entire transcript, 3SEQ creates a sequencing library with one sequencing primer targeting the poly-A tail. Therefore, all transcripts amplified during PCR and incorporated into the sequencing library contain a portion of the poly-A tail with an upstream sequence of approximately 200 bp in length. Sequencing is then performed uni-directionally, toward the poly-A tail. While the priming techniques utilized in standard-RNA-Seq will typically produce multiple reads per single transcript (dependent in large part on the transcript length), the 3SEQ protocol is designed to produce a single read per transcript; therefore, this technique is not susceptible to length-bias, a problem which significantly complicates attempts at gene expression profiling using standard RNA-Seq [49]. Performing appropriate reads-per-gene normalization based on putative transcript length for transcriptional profiling with standard RNA-Seq on degraded RNA would be far more difficult than normalization from high quality starting RNA, since in the setting of severe RNA degradation putative transcript length is irrelevant; consequently, the denominator in the reads per kilobase of exon model per million mapped reads (RPKM) metric, which is a standard technique for normalization for transcriptional profiling from RNA-Seq [44], is unknown. 3SEQ, by contrast, gives one read per transcript molecule, regardless of degradation and regardless of transcript length. This novel protocol for creating a directional sequencing cDNA library targeted to a transcript's 3′ end is the primary feature that distinguishes 3SEQ from standard RNA-Seq and is the feature that allows precise gene expression profiling from samples with degraded RNA.

### Read Mapping, Filtering, and Quantification

For the 3SEQ data, each 25 bp read was mapped to the genome using Eland. Non-uniquely mapping reads and reads with >1 mismatch were discarded. Using the final protocol, we reproducibly obtained just under 50% uniquely mapping reads (out of the total number of reads that passed the Illumina pipeline quality filter). The major source of non-uniquely mapping reads were poly-A sequences, which constituted approximately 20% of all reads generated by 3SEQ. With improvements in the sequencing technology and software provided by Illumina (subsequent to the experiments described in this manuscript), the total number of reads obtained per 3SEQ run have increased from ∼3–6 million in the runs described in this manuscript, to ∼10–15 million on our most recent runs. For FFPET samples, an average of 2 million reads per sample mapped to within 1KB of an annotated gene on the reference genome, and for frozen samples an average of 1.2 million reads per sample mapped to within 1 KB of an annotated gene on the reference genome. For both FFPET and frozen samples, the highest proportion of annotated reads mapped to the 3′ untranslated region (50% of annotated FFPET reads and 41% of annotated frozen reads), followed by the coding exon (14% FFPET and 22% frozen). The remaining reads mapped to intergenic or intronic regions or mapped with incorrect orientation. Transcripts that did not map to intragenic regions with the correct orientation were not included in this analysis. For each sample, transcripts that mapped uniquely within 1KB of a gene annotation on the reference genome were attributed to that gene symbol, leaving ∼27K unique genes with at least 1 read across the 25 sample-preparation type combinations (14 FFPET and 11 frozen samples) in the 3SEQ analysis. We removed genes with less than 25 total reads across the samples, leaving ∼18K genes.

For the HEEBO data, the log base 2 of the normalized red/green ratio was computed for all microarray spots not flagged as low quality (45,561 HEEBO biosequenceIDs). Multiple probes from the same HUGO gene ID were averaged, and HUGO gene IDs with less than 70% valid data were removed, leaving ∼24K genes.

All statistical analyses comparing HEEBO with 3SEQ were limited to the ∼12K common genes included in the filtered 3SEQ and HEEBO data sets. Prior to performing statistical analyses, the samples were centered by subtracting out the sample mean and scaled by dividing by the sample standard deviation. The distribution of the centered and scaled expression values are provided as Figure S1.

### Correlation of Gene Expression Measurements

To compare the ability of 3SEQ and HEEBO to measure gene expression on FFPET reliably, we computed the Spearman correlation of the gene expression measurements from frozen tissue and FFPET for the 7 samples with matched measurements from both tissue preparations profiled on both 3SEQ and HEEBO. In all 7 cases, the 3SEQ measurements showed higher frozen-FFPET correlation than the HEEBO measurements (Table 1) [mean Spearman rho with 3SEQ = 0.76 vs. 0.46 with HEEBO; Wilcoxon p = 0.008]. These findings suggest that 3SEQ is a more robust platform than HEEBO microarray for gene expression profiling from FFPET.

**Table 1:** Correlation of Gene Expression Profiling Measurements From Frozen Tissue and FFPET on Matched Samples.

Sample	3SEQ	HEEBO	p value
**DTF2435**	0.81	0.62	
**DTF2913**	0.74	0.58	
**SFT200**	0.41	0.23	
**SFT3237**	0.83	0.64	
**SFT3524**	0.9	0.3	
**SFT4711**	0.85	0.25	
**SFT4934**	0.75	0.57	
**SFT2162**	0.85	N/A	
**Mean correlation on matched samples**	0.76	0.46	0.008

The table presents the Spearman's rho statistic as a rank-based measure of association of gene expression measurements on frozen vs. FFPET. The first 7 samples contained matched frozen and FFPET measurements on both 3SEQ and HEEBO microarray. The 8th sample contained only matched samples on 3SEQ. The final row of the table shows the mean correlation on matched samples for 3SEQ and HEEBO with the Wilcoxon test p value to assess the significance of the observed difference in mean frozen-FFPET correlation on matched samples profiled with 3SEQ vs. HEEBO.

The FFPET and frozen samples in our analysis were stored for varying periods of time prior to RNA extraction. The storage duration of the FFPET samples ranged from 1 to 8 years, with a median storage time of 5 years. The frozen samples ranged in storage time from 0 to 15 years with a median storage time of 4 years. Information on specimen age was unavailable for 4 samples in the analysis. In our data set, we do not observe any significant association between storage time of the archival tumor tissue and the correlation between frozen and FFPET measurements on either 3SEQ or HEEBO (both p>0.35).

### Assessing Differential Gene Expression

**Figure 2:**
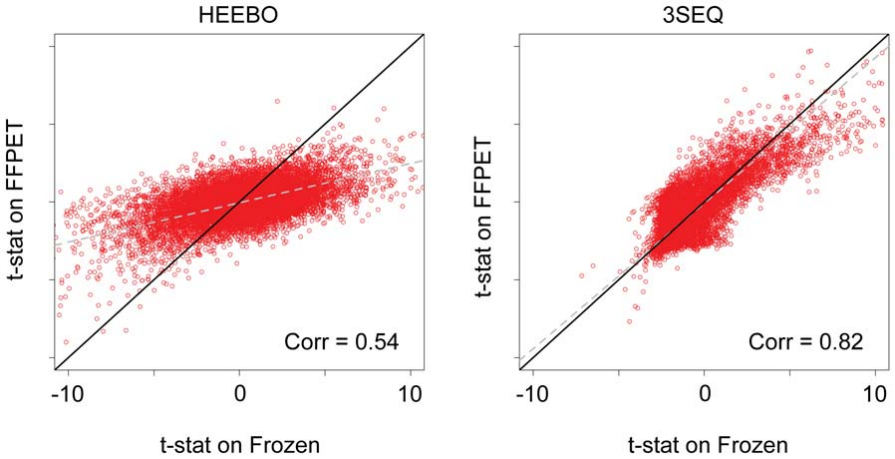
Scatter plot of modified t-statistics on FFPET vs. frozen tissue. Each point is a gene plotted by the t-statistic generated on FFPET vs. the t-statistic generated on frozen tissue. The black line is a line with a slope of 1 and x intercept at 0, corresponding to perfect correlation between the axes. The grey dotted line is a plot of the first principal component. The left plot shows the HEEBO data, and the right plot shows the 3SEQ data.

A primary goal of gene expression profiling studies in cancer research is to identify genes differentially expressed between tumor types [50], and we used this metric as a practical method for evaluation of the performance of the two platforms. For each gene, we computed a modified t-statistic in order to quantify the extent of differential expression between DTF and SFT [51]. We computed the correlation of the test statistic values obtained from gene expression profiling on frozen tissue and FFPET for 3SEQ and HEEBO. This analysis showed a substantially higher correlation of test statistics generated from frozen tissue and FFPET on 3SEQ compared with HEEBO (Pearson correlation = 0.82 on 3SEQ vs. 0.54 on HEEBO, Figure 2). These findings suggest that 3SEQ is superior to HEEBO for obtaining accurate and robust measurements of differential gene expression from FFPET.

**Figure 3:**
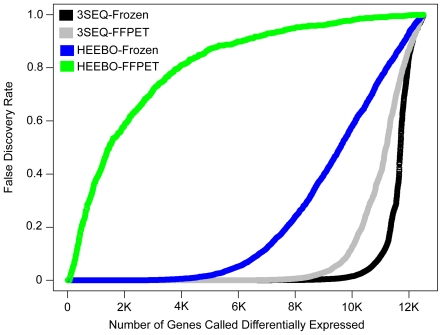
False discovery rate vs. number of genes called significant. The number of genes called differentially expressed between DTF and SFT is plotted along the x axis and the corresponding false discovery rate is plotted along the y axis. The 3SEQ-frozen analysis includes 5 DTF and 6 SFT; the 3SEQ-FFPET includes 6 DTF and 8 SFT; the HEEBO-frozen includes 9 DTF and 8 SFT; and the HEEBO-FFPET includes 6 DTF and 8 SFT.

We used permutations to estimate false discovery rates (FDRs) for the modified t-statistics obtained from the frozen tissue and FFPET for 3SEQ and HEEBO, as described in [52]. Similar FDRs were obtained using 3SEQ on frozen tissue and FFPET. HEEBO microarray resulted in many fewer genes with a low FDR on FFPET as compared with frozen tissue (Figure 3). Using FDR<0.01 as a cutpoint, 9,645 genes were identified as differentially expressed on 3SEQ-frozen, 8,137 on 3SEQ-FFPET, 4,574 on HEEBO-frozen, and only 69 on HEEBO-FFPET (Figure 3, Table S2). These findings demonstrate that in terms of identifying differentially-expressed genes with low FDRs, 3SEQ is far more effective than HEEBO microarray on FFPET.

Different numbers of samples were used for each of the 4 platform-tissue type combinations assessed in the primary analysis in this study. Importantly, the 3SEQ analyses were performed with fewer total samples than the HEEBO analyses. Therefore, we would expect the differential sample sizes to favour HEEBO over 3SEQ. To confirm this hypothesis, we sampled 5 DTF and 6 SFT from each of the platform-tissue type combinations and repeated the analysis with equal sample sizes, and as expected, this showed similar results to those obtained with the full dataset, with a slight improvement in the performance of 3SEQ relative to HEEBO (Figure S2).

### Agreement of Differentially Expressed Gene Lists

**Figure 4:**
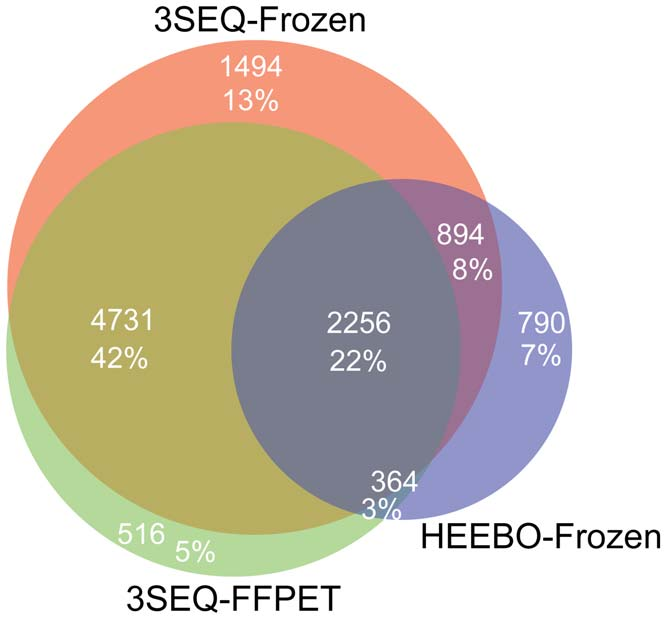
Venn diagram of genes called significant in each platform-tissue type combination at an FDR<0.01. The orange circle includes the set of genes identified as differentially expressed by 3SEQ-frozen, the green circle by 3SEQ-FFPET, and the lavender circle by HEEBO-frozen. Only 69 HEEBO-FFPET genes reached significance at this threshold, and the HEEBO-FFPET gene list was not plotted in the Venn diagram. The number of genes and percentage of total genes in each portion of the Venn diagram are labelled.

We next assessed the agreement of the lists of genes differentially expressed between DTF and SFT (FDR<0.01) (Figure 4). 89% of the genes identified as differentially expressed by 3SEQ on FFPET were also identified as differentially expressed by 3SEQ on frozen tissue. 82% of genes identified as differentially expressed by HEEBO-frozen were also identified as differentially expressed by either 3SEQ-frozen or 3SEQ-FFPET.

### Biological Pathways in DTF and SFT

To assess the biological significance of the statistically significant gene lists, we used the DAVID set of bioinformatics resources [53] to identify the KEGG biological pathways [54] most significantly enriched in the top 1000 ranked genes with relatively higher expression in DTF and the top 1000 genes with relatively higher expression in SFT. Since far fewer than 1000 genes from the HEEBO-FFPET analysis yielded FDR<0.01, we selected the top ranked genes with FDR<0.05 for inclusion in the HEEBO-FFPET gene lists. We performed this analysis separately for each of the gene lists generated from the differential gene expression analysis of each of the 4 platform-tissue type combinations (Table S3 and Table S4).

#### Biological pathways with relatively increased expression in DTF

Functional gene set analysis from all 4 platform-tissue type combinations identified the KEGG pathway ECM-receptor interaction as relatively enriched in DTF. This gene set is comprised of proteins that function in the interaction of cells with extracellular matrix, including integrins (ITGB1, ITGB5), collagens (COL1A1, COL1A2, COL5A1, COL6A2), glycoproteins (FN1, THBS2, SDC1), and other cell-surface-associated components. DTF is a fibroblastic neoplasm, so it was not surprising that this gene set, which contains genes known to be expressed in stromal cells and to play important roles in regulation of the extracellular matrix, was highly expressed in DTF. The fact that this pathway was identified by analysis of HEEBO-FFPET data demonstrates that although very few genes were identified as differentially expressed on HEEBO-FFPET, the set of genes identified as highly expressed in DTF represents a coherent gene set that provides insight into DTF biology. The Wnt signalling pathway is known to play an important role in DTF [55]–[60], and we have previously noted that Wnt pathway genes show increased expression in DTF, based on microarray data from frozen tissue [47], [48]. 2 Wnt-signalling related KEGG pathways (Wnt signalling and melanogenesis) were identified as enriched in DTF only on 3SEQ-frozen and 3SEQ-FFPET, but not on HEEBO-frozen or HEEBO-FFPET in our current functional gene set analysis. The re-identification of WNT-signalling pathways by 3SEQ supports the ability of gene expression profiling by 3SEQ to identify a key oncogenic pathway in DTF not only on frozen tissue but also on FFPET.

Several other pathways were identified as relatively highly expressed in DTF by at least 1 of the platform-tissue type combinations. 3 of these pathways are closely related to and share multiple genes in common with the ECM-receptor interaction pathway (cell communication, focal adhesion, regulation of actin cytoskeleton). 3 other pathways were identified only on HEEBO-frozen (glycan structures - biosynthesis 1, axon guidance, adherens junction) and their significance in DTF must be more fully evaluated in future studies.

#### Biological pathways with relatively increased expression in SFT

Analysis of HEEBO-FFPET data identified no pathways as enriched in SFT. The KEGG pathway “prostate cancer” was identified as relatively highly expressed in SFT by 3SEQ-frozen, 3SEQ-FFPET, and HEEBO-frozen. The genes (FGFR1, BCL2, IGF1, PDGFD, TCF7L2) from this pathway were identified by all 3 platform-tissue type combinations as highly expressed in SFT. It has recently been shown that insulin signalling plays an important role in SFT pathogenesis [61], [62]. The “insulin signalling pathway” was identified as significantly enriched in SFT by both 3SEQ-frozen and 3SEQ-FFPET, but was not identified as enriched by either HEEBO-frozen or HEEBO-FFPET. Analysis of 3SEQ data from both fresh tissue and FFPET revealed several additional biological pathways (acute myeloid leukemia, VEGF signaling pathway, oxidative phosphorylation), which are known to play important roles in oncogenesis in other tumors but whose contribution to SFT pathogenesis has not previously been described. In addition, a large set of closely related cancer-associated pathways (endometrial cancer, non-small cell lung cancer, ErbB signalling, MAPK signalling, melanoma, GnRH signalling) were identified as enriched in SFT on the 3SEQ-FFPET data only. These gene sets share multiple genes in common with each other and with the “prostate cancer” set (including: *AKT2*, *BAD*, *MAP2K2*, *PTEN*, *AKT3*, *PIK3R1*, *CREB3L2*, *CREBBP*, *ERBB2*, *EGFR*). These findings further support the ability of gene expression profiling by 3SEQ to identify coherent biological pathways that may play key roles in tumor pathogenesis from both frozen and FFPET.

### Identification of Genes Expressed Exclusively (or Almost Exclusively) in DTF or SFT

**Figure 5:**
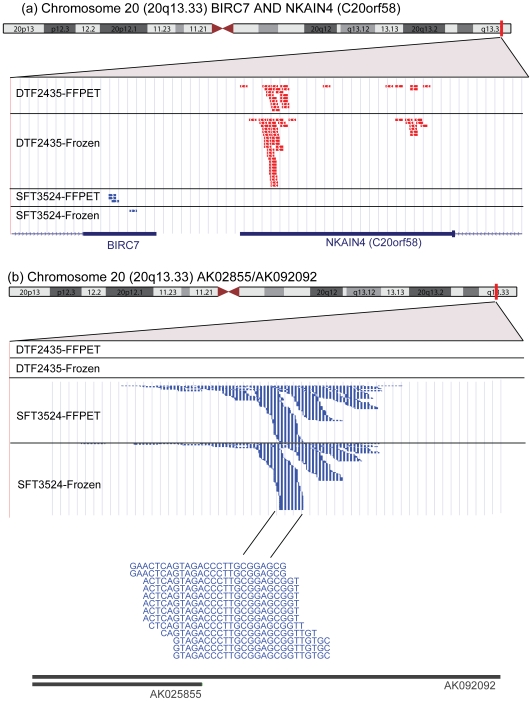
3SEQ reads visualized on the UCSC Genome Browser. The top portion of panels A and B show an ideogram of chromosome 20 with a vertical red bar at cytoband 20q13.33. A small portion of this cytoband is expanded and displays four custom tracks beneath it: DTF2435-FFPET, DTF2435-Frozen, SFT3524-FFPET, and SFT3524-Frozen. Each of these tracks displays the 3SEQ sequencing reads from a single DTF sample (DTF2435) and a single SFT sample (SFT3524), whose gene expression was measured from both FFPET and frozen tissue. Each track displays a red or blue block indicating a 3SEQ read that mapped to the displayed portion of the genome. The blocks are colored according to the read's directionality with reads aligned to the genome in the forward (left to right) direction in blue and reads aligned to the genome in the reverse orientation in red. In panel a, two adjacent genes are displayed on the bottom of the panel with the gene on the left (BIRC7) oriented 5′ to 3′ from left to right, and the gene on the right (NKAIN4/C20orf58) oriented 5′ to 3′ from the right to left. Panel a shows that NKAIN4 is expressed at a moderate level exclusively in DTF (both FFPET and frozen), while BIRC7 shows a total of 4 reads exclusively in the SFT sample (both FFPET and frozen) with no reads in the DTF sample. In this example, all reads mapped with the correct orientation to the 3′ portion of the transcript. Panel B shows a higher magnification display of a nearby region on 20q13.33. This genomic region encodes a transcript (AK025855/AK092092) that is expressed exclusively in SFT3524 (FFPET and Frozen) with no expression in DTF2435. Beneath the display of the piles of reads at the 3′ end of the transcript, a higher magnification view of the actual read sequences from a portion of the pile is displayed.

DTF and SFT are both fibroblastic neoplasms, which may represent tumors composed of different stromal cell types or different pathways of tumor differentiation from a common stromal cell type. To identify additional diagnostic markers and to better understand DTF and SFT pathogenesis, it would be useful to identify genes that show at least low or moderate levels of expression in DTF or SFT with virtually no expression in the other tumor type. This type of analysis is very difficult to perform using expression data from microarrays, since the measurements are complicated by the presence of background hybridization signal making it very difficult to confidently identify genes that show an expression level near zero in either tumor type. In contrast, the data produced by sequencing is discrete with far less background noise than microarray data, which greatly facilitates the identification of genes expressed in only DTF or SFT (Figure 5). For this analysis (which we limited to the 3SEQ data), we started with the ∼18K genes that showed at least 25 reads across all samples. For each gene, we computed the fraction of the DTF reads in the full data set that occur in that gene, and divided this by the fraction of the SFT reads in the full data set that occur in that gene. A high score indicates that the gene is highly expressed in DTF relative to SFT, and a score near zero indicates the opposite. We performed this analysis separately on both the 3SEQ-frozen and 3SEQ-FFPET data (Table S5).

18 genes showed either completely exclusive expression in DTF or at least 100 fold increased expression in DTF compared with SFT on both the 3SEQ-frozen and 3SEQ-FFPET analyses. This list includes genes known to be expressed in muscle (MB, MYH7, TNNI1, TNNT1) and genes involved in development (GJB2, ACAN, DMRT2, SLC5A1, MYOD1, PAX1). These findings suggest that a myofibroblastic phenotype including increased expression of genes involved in developmental processes and expressed in muscle is specific for DTF compared with SFT. In addition to known genes, the list includes several poorly characterized transcripts (C20orf58, DFKZp686J02145), which may provide new insights into DTF pathogenesis.

44 genes showed either completely exclusive expression in SFT (with at least 100 total reads across the samples) or at least 100 fold increased expression in SFT as compared with DTF on both the 3SEQ-frozen and 3SEQ-FFPET analyses. This list includes genes known to be involved in signal transduction (NPW, GRIA2, KNDC1, and NRGN), as well as glycoproteins and genes expressed in the extracellular matrix (PCSK2, FGG, CHGA, MMP3, SFTPB, PYY). In addition, the list contains several poorly characterized transcripts (including C1orf92, AB058691, and LOC126520), which may provide insight into SFT pathogenesis and serve as candidate novel biomarkers.

#### Identification of biological pathways enriched in genes identified as differentially expressed in 3SEQ-frozen but not 3SEQ-FFPET.

To identify biological pathways enriched in the set of ∼2300 genes identified as differentially expressed (FDR<0.01) on 3SEQ-frozen but not 3SEQ-FFPET, we performed a functional gene set analysis using DAVID [54], which showed that the cluster of functional annotation groups most significantly enriched in this gene set were related to intracellular signalling, suggesting that transcripts encoding proteins involved in intracellular signalling may show increased susceptibility to degradation in FFPET.

## Discussion

Since the introduction of gene expression microarrays in the mid-1990s, genome-wide expression profiling has been widely utilized in cancer research [63], [64]. Gene expression profiling experiments have led to significant advances in our understanding of a wide range of human malignancies, but clinical research efforts have been frustrated by lack of specimens. A major hindrance to the translation of gene expression profiling to the clinic is the fact that gene expression microarrays are best performed on fresh frozen tissue, and few samples are stored as fresh frozen. In contrast, essentially all tumor specimens are stored as FFPET [28]. This fixation and storage technique results in extensive RNA fragmentation [29]. Several groups have attempted to use FFPET for gene expression profiling by microarrays [22]–[27] with mixed results. Difficulties of gene expression profiling by microarray on FFPET include the inability to obtain adequate RNA for gene microarray profiling from most archival samples [25] and a lack of sensitivity for identifying genes known to be expressed from frozen tissue in matched FFPET samples [26].

Due to the difficulty of gene expression profiling from FFPET using microarrays, several groups have utilized RT-PCR to quantify the expression of targeted sets of genes [29], [30], [65]. Recently, Hoshida *et al.* performed DASL for gene expression profiling from FFPET and identified a gene expression pattern that correlates with survival in hepatocellular carcinoma [31]; however, this study does not demonstrate the ability for full unbiased genome-wide expression profiling from FFPET, as the analysis was limited to only 6,100 genes targeted by the probe set.

3SEQ is sequencing-based and overcomes the major limitations of hybridization-based expression arrays, allowing more sensitive and precise quantitative measurements for genome-wide expression profiling. The primary feature that differentiates the 3SEQ protocol from standard RNA-Seq protocols is that while standard RNA-Seq typically generates a non-directional sequencing library comprised of fragments of RNA that span the transcript length, the 3SEQ protocol generates a directional sequencing library comprised predominantly of ∼200 bp cDNA fragments with a poly-A tail, in which sequencing will proceed directionally toward the poly-A tail. This novel design is essential for performing accurate transcriptional profiling from severely degraded RNA, because it ensures that one read per transcript molecule is produced, regardless of degradation and regardless of transcript length.

Compared to other existing gene expression profiling methods, the 3SEQ method facilitates gene expression profiling of degraded RNA from FFPET. In the current study, we identified similar numbers of differentially expressed genes between DTF and SFT on frozen tissue (∼9.6K) and FFPET (∼8.1K) by 3SEQ. In contrast, we identified fewer differentially expressed genes on frozen tissue (∼4.6K) and far fewer genes on FFPET (only 69 genes) by HEEBO microarray. These data clearly indicate that, in contrast to HEEBO microarray, 3SEQ is effective for genome-wide gene expression profiling on both frozen tissue and FFPET, with similar performance on the two tissue types.

Functional gene set analysis of 3SEQ data from frozen tissue and FFPET revealed two key sets of pathways (Wnt signalling-related pathways, and extracellular matrix-related pathways) to be enriched in DTF. HEEBO analysis identified the extracellular matrix-related pathways, but failed to identify significant enrichment in the Wnt signalling pathways, which are known to play a critical role in DTF [55]–[60]. In addition, functional gene set analysis of genes with relatively increased expression in SFT by 3SEQ identified insulin signalling as a significantly enriched pathway and recent studies suggest that insulin receptor activation is frequently seen in SFT [61], [62]. Analysis of 3SEQ data from both frozen tissue and FFPET revealed several related cancer-associated pathways (prostate cancer, VEGF signalling, acute myeloid leukemia) containing a number of genes known to be important in carcinogenesis (AKT2, IGF1, BAD, PIK3R1, CCND1, PML, RARA), whose concerted role in SFT has not previously been characterized. There were no pathways identified by HEEBO-FFPET as enriched in SFT. These findings further suggest that 3SEQ is superior to HEEBO microarray for gene expression profiling from FFPET.

An additional advantage of 3SEQ data is that it can be utilized to identify genes expressed in at least low or moderate levels in one of the tumor types with virtually no expression in the other tumor type. This type of quantitative analysis, which is very difficult to perform on microarray data due to background noise and hybridization artefacts, may facilitate a deeper understanding of differential pathways of tumor pathogenesis and the identification of highly specific diagnostic markers.

These findings have significant implications for translational cancer research. A major goal of translational cancer research is to identify diagnostic markers, prognostic markers, and markers to predict response to treatment. Each of these goals requires the acquisition of large numbers of well-annotated clinical specimens with long term follow-up [19]. The lack of adequate samples with detailed clinical information (such as drug response) has been a major impediment to the translation of gene expression profiling findings to the clinic [18]. We believe 3SEQ could revolutionize the field of translational cancer genomics, by allowing investigators to perform gene expression profiling on large numbers of well-annotated archival tumor specimens with long term follow-up. Experiments could then be designed to identify gene expression signatures to predict specific clinically important phenotypes (such as drug response, progression/recurrence risk, and survival) and to gain a deeper understanding of cancer biology.

## Methods

### Ethics Statement

The soft tissue tumors were collected using HIPAA compliant Stanford University Medical Center institutional review board approved written informed consent. Some of the tissues already existed in tissue banks and fall under exemption 4.

### Tumor Samples

The study included a total of 23 samples, which were profiled using at least one of the 4 platform-tissue type combinations (3SEQ-frozen, 3SEQ-FFPET, HEEBO-frozen, HEEBO-FFPET) (Table S1). Overall, the HEEBO-frozen analysis contained 17 samples (DTF n = 9, SFT n = 8), the HEEBO-FFPET contained 14 samples (DTF n = 6,SFT n = 8), 3SEQ-frozen contained 11 samples (DTF n = 5,SFT n = 6), and 3SEQ-FFPET contained 14 samples (DTF n = 6, SFT = 8). The archival samples used in all analyses were collected from 2000–2007. All cases represented classic examples of sporadic type DTF and benign SFT. Additional information on the tumors is provided in Table S1.

### RNA Preparation

For total RNA extraction from frozen tissue stored at −80°C, tissue was homogenized in Trizol reagent (GibcoBRL/Invitrogen, Carlsbad, USA, CAT#15596-018) and total RNA was prepared. For total RNA extraction from FFPET, multiple 20 µm sections were cut from each paraffin block and deparaffinised using xylene and ethanol. A protease digestion was then performed prior to nucleic acid isolation, nuclease digestion, and final RNA purification (Recover All Total Nucleic Acid Isolation kit, Ambion CAT#AM1975).

### Labeling and Hybridization to HEEBO Microarrays

Prior to labelling, the total RNA from frozen samples used for the HEEBO microarray was subject to a DNAse treatment (Qiagen RNAse-Free DNAse Set) and RNA clean-up procedure (RNeasy Mini Kit). The total RNA extracted from FFPET was amplified using a procedure described elsewhere [66]. The HEEBO microarrays contain 44,544 70-mer probes designed using a transcriptome-based annotation of genomic loci (http://www.microarray.org/sfgf/heebo.do). We performed: indirect labelling of the tumor total RNA-derived cDNA (Cy5) and reference RNA-derived cDNA (Cy3); microarray hybridization and washing; and scanning with GenePix 4000 microarray scanner and GenePix software as previously described [67]. All microarray data is MIAME compliant and the raw data has been deposited in the Stanford Microarray Database (http://smd.stanford.edu/) and the Gene Expression Omnibus (http://www.ncbi.nlm.nih.gov/geo/) with accession number GSE18209.

### Generation of 3SEQ Sequencing Library

mRNA was isolated from 10 µg of total RNA extracted from either frozen tissue or FFPET by polyA selection using Oligotex mRNA Kit (Qiagen). For mRNA from frozen tissue, RNA heat fragmentation was combined with heat denature in cDNA synthesis: 9 µl of mRNA was fragmented to 100–200 bases by incubation with 4 µl of First Strand Buffer and 1 µl of 100 µM oligo-dT_P7 RT primer at 85°C for 10 minutes; RNA fragmentation was assessed by gel electrophoresis; the mixture was then cooled down to 50°C and followed by the 1^st^ strand cDNA synthesis in 20 µl reaction with Superscript III Reverse Transcriptase (Invitrogen) for 1 hour at 50°C; the 1^st^ strand cDNA was then converted to double-stranded cDNA and end repaired as described by the manufacture. The mRNA from FFPET was converted directly to cDNA without any fragmentation using the procedure as described above for frozen tissue with the exception that the 4 µl of First Strand Buffer was added to the reaction after the 85°C denature step and the reaction has been cooled down to 50°C. After purification with Qiagen MinElute Kit, the cDNA was ligated to double-stranded P5 Linker (1 µl of 100 µM) overnight at 16°C. The linker-ligated cDNA was purified and size selected for 200–300 bp fragment by agarose gel fractionation. The selected linker-ligated cDNA contains P7 sequence at the 3′ end immediately downstream of poly A and P5 at the 5′ end. The final library was generated by PCR amplification of the Linker-ligated cDNA with two primers of P5 and P7 and Phusion PCR Master Mix (New England Biolab) using a 15 cycle program (98°C for 30 sec; 15 cycles of 98°C for 10 sec, 65°C for 30 sec, 72°C for 30 sec; 72°C for 5 min).

### Sequencing and Read Mapping on the Genome

Following generation of the sequencing library, sequencing was performed using the Illumina Genome Analyzer II.

### Gene Filtering, Scaling, and Quantification

For the HEEBO data, the log base 2 of the normalized red/green ratio was computed for all microarray spots not flagged as low quality (45,561 HEEBO biosequenceIDs). Multiple probes from the same HUGO gene ID were averaged, and HUGO gene IDs with less than 70% valid data were removed, leaving ∼24K genes.

For the 3SEQ data, each 25 bp read was mapped to the genome (Hg18) using Eland software, provided by Solexa. Non-unique hits and hits with >1 mismatch were discarded. For each sample, the sum of the unique annotated hits located within 1KB of a gene annotation on the reference genome was computed, leaving ∼27K genes with at least 1 hit. We removed genes with less than 25 total hits across 25 samples, leaving ∼18K genes.

Statistical analyses comparing 3SEQ and HEEBO were limited to the ∼12K genes included in the filtered 3SEQ data set and present on the HEEBO microarray platform.

### Statistical Analysis

#### Data centering and scaling

All samples were centered by subtracting the sample mean and scaled by dividing by the sample standard deviation. All statistical analyses comparing 3SEQ and HEEBO were performed separately on the scaled measurements from the 4 platform-tissue preparation combinations (3SEQ-frozen, 3SEQ-FFPET, HEEBO-frozen, HEEBO-FFPET).

#### Correlation of gene expression measurements

To assess the correlation of gene expression measurements from frozen tissue and FFPET on 3SEQ and HEEBO, we computed Spearman's rho as a rank-based non-parametric measure of correlation. To assess the correlation of the test statistics generated on frozen tissue and FFPET, we computed the Pearson's correlation of the test statistics on each of the platforms.

*Significance testing:* Significance testing was performed using modified two-sample t-statistics in order to identify genes differentially expressed between DTF and SFT. FDRs were estimated by permutations. The modified t-statistic was computed as described in [51] as: 
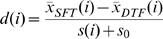
, where 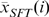 and 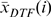 are defined as the average expression levels for gene 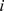 in SFT and DTF respectively, 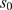 is a small positive constant, which we set to 0.05, and 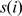 is the standard deviation of repeated expression measurements. To generate an FDR for symmetric cut-offs of the test statistics, we: permuted the sample class labels for 150 iterations; computed a modified t-statistic (as described above) for each gene during each iteration; and calculated the FDR for test statistic *c* as: 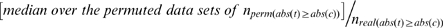, where 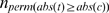 is the number of genes in each permuted dataset with a t-statistic greater than or equal to *c* in absolute value, and 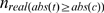 is the number of genes in the real dataset with a t-statistic greater than or equal to *c* in absolute value.

#### Functional gene set analysis

Functional gene set analysis was performed using the DAVID set of bioinformatics tools [53], [68]. For the gene set analysis, we ranked the genes by test statistic and submitted the top 1000 genes in DTF and SFT for 3SEQ-frozen, 3SEQ-FFPET, and HEEBO-frozen. For HEEBO-FFPET we submitted the genes relatively highly expressed in DTF (114 genes) and SFT (57 genes) with an FDR<0.05. We identified the KEGG pathways containing at least 10 genes from the submitted gene list with a modified Fisher exact p value (EASE score [53]) less than 0.05.

## Supporting Information

Figure S1 Histograms of probability density of scaled and centered expression values. (A) 3SEQ-FFPET; (B) 3SEQ-frozen; (C) HEEBO-FFPET; and (D) HEEBO-frozen. The x axis is the scaled expression value. The y axis is the histogram density.
(0.34 MB TIF)

Figure S2 False discovery rate vs. number of genes called significant with equal sample sizes. Since our dataset contained slightly different numbers of samples in each platform-tissue type combination (Table S1), we performed the FDR vs. genes called significant analysis with all platform-tissue type combinations containing 5 DTF vs. 6 SFT samples. For this analysis, we performed 50 iterations, in which we selected 5 DTF and 6 SFT samples from each platform-tissue type combination. We then took the mean and median of the results across the 50 iterations. The mean (A) and median (B) results with equal sample sizes of the FDR vs. number of genes differentially expressed plots are displayed.
(0.99 MB TIF)

Table S1 DTF and SFT tumor samples included in the analysis. The table lists the 23 sample IDs in the first column. The next 3 columns list the tumor resection year, RNA extraction year, and RNA yield, respectively. The final 4 columns are labelled 3SEQ-FFPET, 3SEQ-Frozen, HEEBO-FFPET, and HEEBO-Frozen, respectively. A cell contains an “X” if the row's sample was profiled by the platform-tissue type combination indicated by the column's header.
(0.06 MB DOC)

Table S2 Test statistics and FDRs. The first column indicates the gene symbol for the 12,512 genes included in the analysis. The next 8 columns display the row's gene's modified t-statistic and FDR obtained by 3SEQ-frozen, 3SEQ-FFPET, HEEBO-frozen, and HEEBO-FFPET, respectively. A negative t-statistic indicates that the gene showed relatively higher expression in DTF, and a positive t-statistic indicates that the gene showed relatively higher expression in SFT.
(2.37 MB XLS)

Table S3 Summary of results from functional gene set analysis. All 25 KEGG gene sets that showed relative enrichment in either DTF or SFT by 1 of the 4 platform-tissue type combinations is indicated in the first column. The next 8 columns contain an “X” if the row's KEGG biological pathway was identified as relatively enriched in DTF (columns B–E) or SFT (columns F–I) by analysis on 3SEQ-frozen (columns B and F), 3SEQ-FFPET (columns C and G), HEEBO-frozen (columns D and H), and HEEBO-FFPET (columns E and I).
(0.06 MB DOC)

Table S4 Detailed results of functional gene set analysis. This table displays separately the results from 3SEQ-frozen, 3SEQ-FFPET, HEEBO-frozen, and HEEBO-FFPET for KEGG biological pathways identified as relatively highly expressed in DTF or SFT. Each enriched KEGG biological pathway is indicated in column B, the numbers of genes from the pathway differentially expressed in DTF or SFT is presented in column C, the modified Fisher exact p-value (EASE score) for the enrichment is presented in column D, and the genes from the pathway identified as highly expressed in DTF or SFT are provided in column E.
(0.08 MB DOC)

Table S5 Genes expressed exclusively (or almost exclusively) in DTF or SFT. This list presents the 44 genes identified as exclusively (or almost exclusively) expressed in SFT (A) or DTF (B) in the analysis of the 3SEQ data. The criteria for inclusion on this list was the gene must show at least 100 reads across the DTF or SFT samples and be expressed exclusively in DTF or SFT or show at least 100 fold increased expression in DTF or SFT.
(0.07 MB DOC)
