# Supplementary figures and images for: 3′-End Sequencing for Expression Quantification (3SEQ) from Archival Tumor Samples
Source: PLoS One. 2010 Jan 19;5(1):e8768. doi: 10.1371/journal.pone.0008768 (PMC2808244; doi:10.1371/journal.pone.0008768)

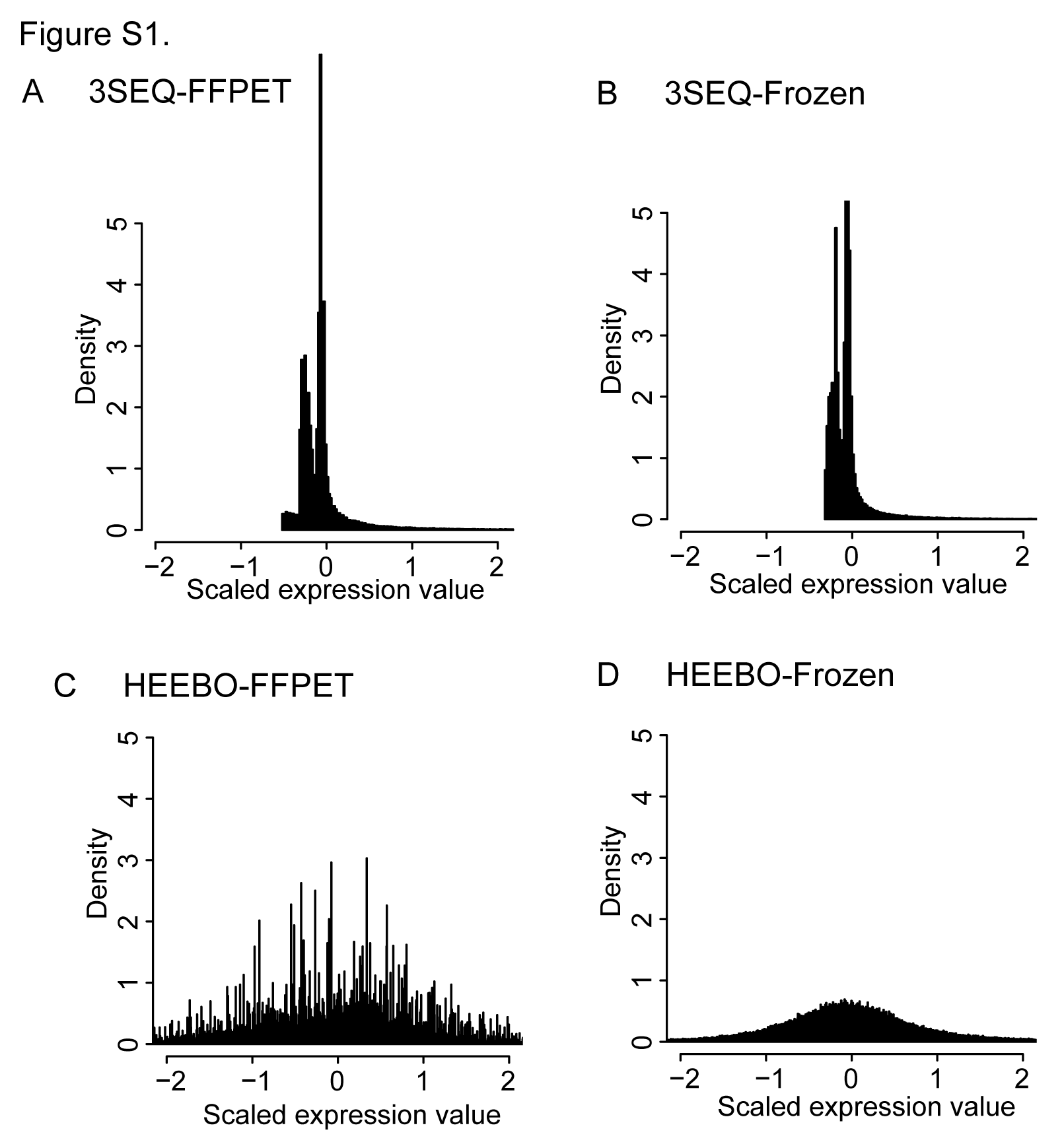

Supplement: Figure S1 — Histograms of probability density of scaled and centered expression values. (A) 3SEQ-FFPET; (B) 3SEQ-frozen; (C) HEEBO-FFPET; and (D) HEEBO-frozen. The x axis is the scaled expression value. The y axis is the histogram density. (0.34 MB TIF) [file pone.0008768.s001.tif]

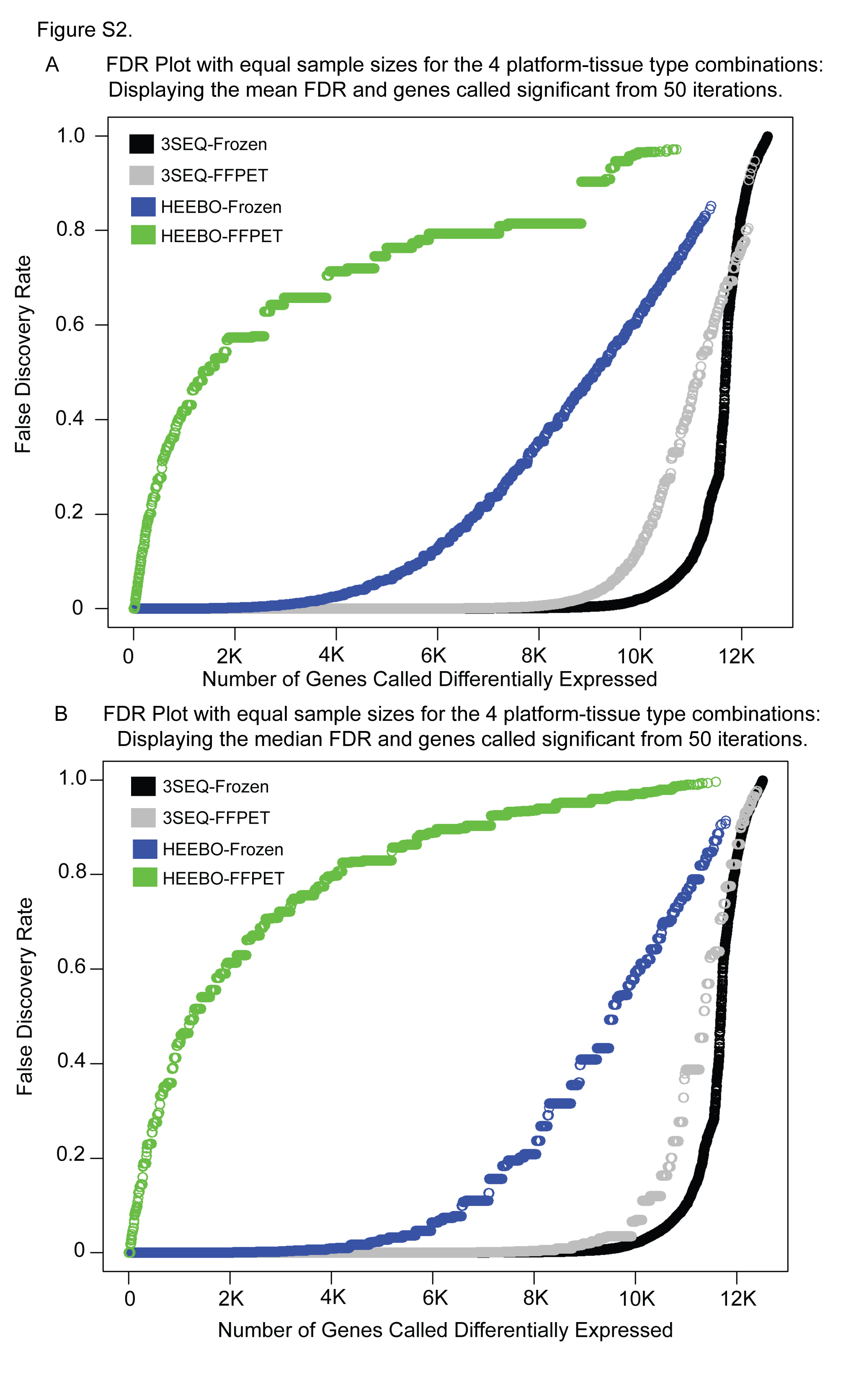

Supplement: Figure S2 — False discovery rate vs. number of genes called significant with equal sample sizes. Since our dataset contained slightly different numbers of samples in each platform-tissue type combination (Table S1), we performed the FDR vs. genes called significant analysis with all platform-tissue type combinations containing 5 DTF vs. 6 SFT samples. For this analysis, we performed 50 iterations, in which we selected 5 DTF and 6 SFT samples from each platform-tissue type combination. We then took the mean and median of the results across the 50 iterations. The mean (A) and median (B) results with equal sample sizes of the FDR vs. number of genes differentially expressed plots are displayed. (0.99 MB TIF) [file pone.0008768.s002.tif]
