# Supplementary material for: 3′-End Sequencing for Expression Quantification (3SEQ) from Archival Tumor Samples
Source: PLoS One. 2010 Jan 19;5(1):e8768. doi: 10.1371/journal.pone.0008768 (PMC2808244; doi:10.1371/journal.pone.0008768)
Supplement: Table S1 — DTF and SFT tumor samples included in the analysis. The table lists the 23 sample IDs in the first column. The next 3 columns list the tumor resection year, RNA extraction year, and RNA yield, respectively. The final 4 columns are labelled 3SEQ-FFPET, 3SEQ-Frozen, HEEBO-FFPET, and HEEBO-Frozen, respectively. A cell contains an “X” if the row's sample was profiled by the platform-tissue type combination indicated by the column's header. (0.06 MB DOC) [file pone.0008768.s003.doc]

Table S1. Samples profiled by each platform-tissue type combination.

| **CASE.ID** | **TUMOR RESECTION**  **YEAR** | **RNA**  **EXTRACTION YEAR** | **RNA YIELD (µg)** | **3SEQ-FFPET** | **3SEQ-Frozen** | **HEEBO-FFPET** | **HEEBO-**  **Frozen** |
| --- | --- | --- | --- | --- | --- | --- | --- |
| DTF1968 | 2001 | 2004 | Frozen: 464 |  |  |  | X |
| DTF1969 | 2001 | 2004 | Frozen: 500 |  |  |  | X |
| DTF1986 | 2000 | 2004 | Frozen: 307 |  |  |  | X |
| DTF2435 | 2001 | Frozen: 2006 FFPET: 2006 | Frozen: 820  FFPET: 24 | X | X | X | X |
| DTF2913 | 2002 | Frozen: 2005 FFPET: 2006 | Frozen: 482  FFPET: 28 | X | X | X | X |
| DTF3119 |  | 2004 | Frozen: 367 |  | X |  | X |
| DTF3120 |  | 2004 | Frozen: 1014 |  |  |  | X |
| DTF3122 |  | 2004 | Frozen: 718 |  | X |  | X |
| DTF3125 |  | 2004 | Frozen: 753 |  | X |  | X |
| DTF4372 | 2003 | 2009 | FFPET: 40 | X |  | X |  |
| DTF5385 | 2007 | 2009 | FFPET: 52 | X |  | X |  |
| DTF5386 | 2006 | 2009 | FFPET: 34 | X |  | X |  |
| DTF5387 | 2007 | 2009 | FFPET: 40 | X |  | X |  |
| SFT1079 | 2001 | 2009 | FFPET: 40 | X |  | X | X |
| SFT1637 | 2001 | 2009 | Frozen: 780 |  |  |  | X |
| SFT200 | 2000 | Frozen: 2004 FFPET: 2008 | Frozen: 151  FFPET: 22 | X | X | X | X |
| SFT2162 | 2001 | Frozen: 2006 FFPET: 2009 | Frozen: 68  FFPET: 66 | X | X | X |  |
| SFT2774 | 1989 | 2004 | Frozen: 421 |  |  |  | X |
| SFT3237 | 2002 | Frozen: 2006 FFPET: 2007 | Frozen: 300  FFPET: 40 | X | X | X | X |
| SFT3524 | 2002 | Frozen: 2005 FFPET: 2007 | Frozen: 522  FFPET: 44 | X | X | X | X |
| SFT4711 | 2005 | Frozen: 2006 FFPET: 2007 | Frozen: 424  FFPET: 44 | X | X | X | X |
| SFT4934 | 2006 | Frozen: 2006 FFPET: 2007 | Frozen: 1700  FFPET: 26 | X | X | X | X |
| SFT5388 | 2001 | 2001 | FFPET: 29 | X |  | X |  |
