# Supplementary material for: 3′-End Sequencing for Expression Quantification (3SEQ) from Archival Tumor Samples
Source: PLoS One. 2010 Jan 19;5(1):e8768. doi: 10.1371/journal.pone.0008768 (PMC2808244; doi:10.1371/journal.pone.0008768)
Supplement: Table S3 — Summary of results from functional gene set analysis. All 25 KEGG gene sets that showed relative enrichment in either DTF or SFT by 1 of the 4 platform-tissue type combinations is indicated in the first column. The next 8 columns contain an “X” if the row's KEGG biological pathway was identified as relatively enriched in DTF (columns B–E) or SFT (columns F–I) by analysis on 3SEQ-frozen (columns B and F), 3SEQ-FFPET (columns C and G), HEEBO-frozen (columns D and H), and HEEBO-FFPET (columns E and I). (0.06 MB DOC) [file pone.0008768.s005.doc]

**Table S3**. Summary of results from functional gene set analysis.

|  | **DTF** | | | | **SFT** | | | |
| --- | --- | --- | --- | --- | --- | --- | --- | --- |
|  | **3SEQ** | | **HEEBO** | | **3SEQ** | | **HEEBO** | |
|  | **Frozen** | **FFPET** | **Frozen** | **FFPET** | **Frozen** | **FFPET** | **Frozen** | **FFPET** |
| ECM-receptor interaction | X | X | X | X |  |  |  |  |
| Cell Communication | X |  | X | X |  |  |  |  |
| Focal adhesion | X |  | X | X |  | X |  |  |
| Melanogenesis | X | X |  |  |  |  |  |  |
| Wnt signaling pathway | X | X |  |  |  |  |  |  |
| Regulation of actin cytoskeleton | X |  | X |  |  |  |  |  |
| Basal cell carcinoma | X |  |  |  |  |  |  |  |
| Glycan structures - biosynthesis 1 |  |  | X |  |  |  |  |  |
| Axon guidance |  |  | X |  |  |  |  |  |
| Adherens junction |  |  | X |  |  | X |  |  |
| Endometrial cancer |  |  |  |  |  | X |  |  |
| Non-small cell lung cancer |  |  |  |  |  | X |  |  |
| Chronic myeloid leukemia |  |  |  |  |  | X |  |  |
| Tight junction |  |  |  |  |  | X |  |  |
| ErbB signaling pathway |  |  |  |  |  | X |  |  |
| MAPK signaling pathway |  |  |  |  |  | X |  |  |
| Melanoma |  |  |  |  |  | X |  |  |
| Calcium signaling pathway |  |  |  |  |  |  | X |  |
| GnRH signaling pathway |  |  |  |  |  | X |  |  |
| Insulin signaling pathway |  |  |  |  | X | X |  |  |
| VEGF signaling pathway |  |  |  |  | X | X |  |  |
| Long-term potentiation |  |  |  |  |  | X | X |  |
| Oxidative phosphorylation |  |  |  |  | X | X |  |  |
| Acute myeloid leukemia |  |  |  |  | X | X |  |  |
| Prostate cancer |  |  |  |  | X | X | X |  |
