# Supplementary material for: 3′-End Sequencing for Expression Quantification (3SEQ) from Archival Tumor Samples
Source: PLoS One. 2010 Jan 19;5(1):e8768. doi: 10.1371/journal.pone.0008768 (PMC2808244; doi:10.1371/journal.pone.0008768)
Supplement: Table S4 — Detailed results of functional gene set analysis. This table displays separately the results from 3SEQ-frozen, 3SEQ-FFPET, HEEBO-frozen, and HEEBO-FFPET for KEGG biological pathways identified as relatively highly expressed in DTF or SFT. Each enriched KEGG biological pathway is indicated in column B, the numbers of genes from the pathway differentially expressed in DTF or SFT is presented in column C, the modified Fisher exact p-value (EASE score) for the enrichment is presented in column D, and the genes from the pathway identified as highly expressed in DTF or SFT are provided in column E. (0.08 MB DOC) [file pone.0008768.s006.doc]

Table S4. Detailed results of functional gene set analysis.

| 3SEQ-Frozen: Relatively enriched in DTF |  |  |  |  |
| --- | --- | --- | --- | --- |
| Category | Term | Count | p Value | Genes |
| KEGG_PATHWAY | hsa04916:Melanogenesis | 11 | 0.03 | FZD1, FZD9, FZD3, CAMK2A, WNT10A, WNT4, GNAI3, DCT, ADCY1, WNT5A, WNT9A, |
| KEGG_PATHWAY | hsa04512:ECM-receptor interaction | 14 | < 0.01 | COL3A1, GP6, COL6A2, SDC1, ITGA11, ITGB1, COL5A1, ITGB3, ITGB5, THBS2, HMMR, COL1A2, COL1A1, THBS1, |
| KEGG_PATHWAY | hsa04310:Wnt signaling pathway | 15 | 0.02 | NKD1, VANGL2, FZD9, WNT5A, SFRP2, RAC3, FZD1, FZD3, CAMK2A, WNT10A, VANGL1, NKD2, AXIN2, WNT4, WNT9A, |

| 3SEQ-Frozen: Relatively enriched in SFT |  |  |  |  |
| --- | --- | --- | --- | --- |
| Category | Term | Count | p Value | Genes |
| KEGG_PATHWAY | hsa00190:Oxidative phosphorylation | 25 | < 0.01 | ATP6V0D1, COX4I1, NDUFV3, NDUFA3, COX5B, NDUFS3, COX7A1, NDUFS7, UQCRC1, ATP6V0B, CYC1, NDUFV1, NDUFB11, NDUFB10, TCIRG1, ATP5D, NDUFS8, NDUFB7, NDUFA11, ATP5I, COX6B1, NDUFA13, UQCR, ATP6AP1, NDUFS6, |
| KEGG_PATHWAY | hsa05215:Prostate cancer | 13 | 0.01 | AKT2, FGFR1, BAD, MAP2K2, BCL2, IGF1, PIK3R1, AKT1, PDGFD, GSTP1, CCND1, TCF7L2, CREB3, |
| KEGG_PATHWAY | hsa04910:Insulin signaling pathway | 16 | 0.04 | AKT2, PRKAR1B, BAD, MAP2K2, RPS6KB2, EXOC7, PIK3R1, AKT1, FLOT2, PFKL, MKNK2, TSC2, PHKG2, CALM3, PRKACB, PRKACA, |
| KEGG_PATHWAY | hsa04370:VEGF signaling pathway | 11 | 0.02 | AKT2, MAPK11, BAD, PPP3CA, PLCG1, SPHK1, MAP2K2, MAPKAPK2, NFATC4, PIK3R1, AKT1, |
| KEGG_PATHWAY | hsa05221:Acute myeloid leukemia | 13 | < 0.01 | AKT2, PML, BAD, RPS6KB2, MAP2K2, JUP, PIK3R1, ZBTB16, RUNX1, AKT1, RARA, CCND1, TCF7L2, |

| 3SEQ-FFPET: Relatively enriched in DTF |  |  |  |  |
| --- | --- | --- | --- | --- |
| Category | Term | Count | p Value | Genes |
| KEGG_PATHWAY | hsa04916:Melanogenesis | 11 | 0.03 | FZD1, FZD9, FZD3, CAMK2A, WNT10A, WNT4, GNAI3, DCT, ADCY1, WNT5A, WNT9A, |
| KEGG_PATHWAY | hsa04512:ECM-receptor interaction | 14 | < 0.01 | COL3A1, GP6, COL6A2, SDC1, ITGA11, ITGB1, COL5A1, ITGB3, ITGB5, THBS2, HMMR, COL1A2, COL1A1, THBS1, |
| KEGG_PATHWAY | hsa04310:Wnt signaling pathway | 15 | 0.02 | NKD1, VANGL2, FZD9, WNT5A, SFRP2, RAC3, FZD1, FZD3, CAMK2A, WNT10A, VANGL1, NKD2, AXIN2, WNT4, WNT9A, |

| 3SEQ-FFPET: Relatively enriched in SFT |  |  |  |  |
| --- | --- | --- | --- | --- |
| Category | Term | Count | p Value | Genes |
| KEGG_PATHWAY | hsa04912:GnRH signaling pathway | 14 | 0.01 | MAP2K4, GNAQ, MAP2K2, JUN, MMP2, ITPR1, MAPK11, PLCB1, MAP2K7, EGFR, RAF1, PRKACB, ITPR2, PRKACA, |
| KEGG_PATHWAY | hsa04910:Insulin signaling pathway | 20 | < 0.01 | PRKAR1B, AKT2, BAD, CBLB, MAP2K2, RPS6KB2, AKT3, PIK3R1, FLOT2, PFKL, PHKB, PKM2, TSC2, INSR, PDE3A, RAF1, PRKACA, PRKACB, GYS1, SORBS1, |
| KEGG_PATHWAY | hsa04510:Focal adhesion | 29 | < 0.01 | ILK, ZYX, ACTN4, PTEN, IGF1, COL6A3, ERBB2, FYN, EGFR, LAMB2, DOCK1, FLNB, LAMB1, AKT2, LAMA2, BAD, FARP2, PIP5K1C, BCL2, COL4A2, AKT3, PIK3R1, JUN, PTK2, ACTB, VWF, CCND3, PDGFD, RAF1, |
| KEGG_PATHWAY | hsa05215:Prostate cancer | 17 | < 0.01 | AKT2, FGFR1, BAD, MAP2K2, PTEN, BCL2, IGF1, AKT3, PIK3R1, CREB3L2, CREBBP, PDGFD, ERBB2, GSTP1, EGFR, RAF1, TCF7L2, |
| KEGG_PATHWAY | hsa04370:VEGF signaling pathway | 12 | 0.01 | AKT2, MAPK11, BAD, PPP3CA, MAP2K2, NFAT5, MAPKAPK2, NFATC4, AKT3, PTK2, RAF1, PIK3R1, |
| KEGG_PATHWAY | hsa05213:Endometrial cancer | 11 | < 0.01 | AKT2, ILK, BAD, ERBB2, MAP2K2, PTEN, AKT3, RAF1, PIK3R1, EGFR, TCF7L2, |
| KEGG_PATHWAY | hsa04720:Long-term potentiation | 12 | < 0.01 | ITPR1, PLCB1, PPP3CA, GNAQ, MAP2K2, GRIA2, RAF1, PRKACA, ITPR2, PRKACB, RPS6KA3, CREBBP, |
| 3SEQ-FFPET: Relatively enriched in SFT (continued) |  |  |  |  |
| KEGG_PATHWAY | hsa00190:Oxidative phosphorylation | 16 | 0.02 | TCIRG1, ATP6V0D1, ATP5D, ATP6V0A1, COX4I1, NDUFS8, NDUFA3, NDUFB7, NDUFA11, ATP5G2, COX6B1, NDUFA13, UQCRC1, CYC1, ATP6AP1, NDUFV1, |
| KEGG_PATHWAY | hsa05223:Non-small cell lung cancer | 10 | 0.01 | AKT2, RASSF1, BAD, ERBB2, MAP2K2, AKT3, RAF1, PIK3R1, EGFR, RXRB, |
| KEGG_PATHWAY | hsa05221:Acute myeloid leukemia | 12 | < 0.01 | AKT2, BAD, PML, MAP2K2, RPS6KB2, RARA, AKT3, RUNX1, RAF1, ZBTB16, PIK3R1, TCF7L2, |
| KEGG_PATHWAY | hsa05220:Chronic myeloid leukemia | 11 | 0.03 | AKT2, CTBP1, BAD, CBLB, MAP2K2, GAB2, AKT3, RUNX1, RAF1, PIK3R1, ABL1, |
| KEGG_PATHWAY | hsa04530:Tight junction | 18 | 0.01 | SYMPK, RRAS, AKT2, ACTN4, CLDN5, PTEN, CASK, EXOC4, AKT3, ACTB, CSNK2B, SPTAN1, ASH1L, MPDZ, GNAI2, PARD3, EXOC3, PPP2R4, |
| KEGG_PATHWAY | hsa04012:ErbB signaling pathway | 15 | < 0.01 | AKT2, MAP2K4, BAD, CBLB, MAP2K2, RPS6KB2, AKT3, JUN, PIK3R1, PTK2, ABL1, MAP2K7, ERBB2, EGFR, RAF1, |
| KEGG_PATHWAY | hsa04010:MAPK signaling pathway | 30 | < 0.01 | ECSIT, RRAS, PPP5C, FGFR1, RPS6KA3, MAPK11, CACNA2D1, MAP2K7, NFATC4, EGFR, TNFRSF1A, FLNB, AKT2, MAP2K4, TAOK1, MAP2K2, MAP2K5, MAPKAPK2, AKT3, MAP4K4, JUN, PTPN7, MAP4K3, PPP3CA, RAF1, MAPK8IP3, PRKACA, PRKACB, TAOK3, MAP3K12, |
| KEGG_PATHWAY | hsa04520:Adherens junction | 15 | < 0.01 | FGFR1, ACTN4, FARP2, CSNK2B, ACTB, CREBBP, ERBB2, INSR, FYN, BAIAP2, WASF2, EGFR, PARD3, TCF7L2, SORBS1, |
| KEGG_PATHWAY | hsa05218:Melanoma | 11 | 0.02 | AKT2, FGFR1, BAD, PDGFD, MAP2K2, PTEN, IGF1, AKT3, RAF1, PIK3R1, EGFR, |

| HEEBO-Frozen: Relatively enriched in DTF |  |  |  |  |
| --- | --- | --- | --- | --- |
| Category | Term | Count | p Value | Genes |
| KEGG_PATHWAY | hsa04520:Adherens junction | 10 | 0.04 | SNAI1, ACTN4, RHOA, RAC3, PTPRM, CDC42, TGFBR1, ACTB, PVRL2, IQGAP1, |
| KEGG_PATHWAY | hsa01430:Cell Communication | 20 | < 0.01 | GJA1, COL6A1, COL6A2, LMNA, ACTB, COL5A1, FN1, LAMC1, GJB2, TNC, THBS2, LAMA4, COL1A2, DSC2, DSG2, COL5A2, COL1A1, LAMB2, DES, THBS1, |
| KEGG_PATHWAY | hsa04512:ECM-receptor interaction | 22 | < 0.01 | ITGA10, COL6A1, COL6A2, SDC1, SDC3, ITGA11, ITGB1, COL5A1, ITGB5, LAMC1, ITGB3, FN1, TNC, THBS2, ITGA5, LAMA4, COL1A2, COL5A2, COL1A1, LAMB2, SDC2, THBS1, |
| KEGG_PATHWAY | hsa04810:Regulation of actin cytoskeleton | 26 | < 0.01 | ACTN4, PDGFA, ITGA10, LIMK1, ARPC2, ITGA11, ARPC1B, PIK3CD, MYH9, MSN, ACTA1, F2R, ITGB1, ACTB, ITGB3, FN1, ITGB5, CFL1, IQGAP1, ITGA5, ACTG2, RHOA, RAC3, CDC42, PFN1, FGF18, |
| KEGG_PATHWAY | hsa04510:Focal adhesion | 34 | < 0.01 | ILK, ACTN4, PDGFA, ITGA10, ITGA11, VEGFB, PIK3CD, LAMC1, TNC, LAMA4, COL1A2, LAMB2, THBS1, PARVB, COL6A1, COL6A2, PARVA, FLNA, ITGB1, ACTB, COL5A1, ITGB5, ITGB3, TLN1, FN1, THBS2, FLNC, ITGA5, RHOA, RAC3, COL5A2, CDC42, COL1A1, PGF, |
| KEGG_PATHWAY | hsa01030:Glycan structures - biosynthesis 1 | 18 | < 0.01 | FUT8, MAN1C1, CHPF, XYLT1, GALNAC4S-6ST, GALNT2, CHST6, GALNTL1, MAN2A1, B4GALT2, B4GALT1, HS3ST3A1, HS3ST3B1, CHSY1, GALNT10, MGAT5, RPN2, CHST1, |
| KEGG_PATHWAY | hsa04360:Axon guidance | 22 | < 0.01 | SEMA3C, PLXNB1, PLXNA1, PLXNC1, LIMK1, NFATC1, ITGB1, GNAI3, SRGAP3, CFL1, PLXNA2, RHOA, RAC3, EFNA4, CDC42, SEMA3B, DPYSL2, GNAI2, CXCL12, EPHB4, PLXNB3, EFNA5, |

| HEEBO-Frozen: Relatively enriched in SFT |  |  |  |  |
| --- | --- | --- | --- | --- |
| Category | Term | Count | p Value | Genes |
| KEGG_PATHWAY | hsa05215:Prostate cancer | 11 | 0.02 | CASP9, FGFR1, PDPK1, ERBB2, BRAF, PDGFD, IGF1, BCL2, EGFR, CREB3L4, TCF7L2, |
| KEGG_PATHWAY | hsa04720:Long-term potentiation | 11 | < 0.01 | ITPR1, RPS6KA6, BRAF, PLCB1, PPP3CA, GNAQ, GRIA2, CAMK4, ITPR2, PRKACB, RPS6KA3, |
| KEGG_PATHWAY | hsa04020:Calcium signaling pathway | 17 | 0.04 | TACR3, ADCY3, GNAQ, PHKA1, ATP2B1, PHKA2, SLC8A1, ITPR1, ERBB2, PPP3CA, PLCB1, ATP2B4, EGFR, CAMK4, PRKACB, ITPR2, PLCE1, |

| HEEBO-FFPET Relatively enriched in DTF |  |  |  |  |
| --- | --- | --- | --- | --- |
| Category | Term | Count | p Value | Genes |
| KEGG_PATHWAY | hsa01430:Cell Communication | 10 | < 0.01 | THBS2, GJA1, COL1A2, COL6A1, COL5A2, COL6A2, COL1A1, ACTB, COL5A1, FN1, |
| KEGG_PATHWAY | hsa04512:ECM-receptor interaction | 10 | < 0.01 | THBS2, COL1A2, COL6A1, COL5A2, COL6A2, COL1A1, ITGB1, COL5A1, FN1, ITGB5, |
| KEGG_PATHWAY | hsa04510:Focal adhesion | 13 | < 0.01 | COL6A2, COL6A1, PARVA, ITGB1, COL5A1, ACTB, ITGB5, FN1, FLNC, THBS2, COL1A2, COL5A2, COL1A1, |

| HEEBO-FFPET Relatively enriched in SFT |
| --- |
| None. |
