# Supplementary material for: 3′-End Sequencing for Expression Quantification (3SEQ) from Archival Tumor Samples
Source: PLoS One. 2010 Jan 19;5(1):e8768. doi: 10.1371/journal.pone.0008768 (PMC2808244; doi:10.1371/journal.pone.0008768)
Supplement: Table S5 — Genes expressed exclusively (or almost exclusively) in DTF or SFT. This list presents the 44 genes identified as exclusively (or almost exclusively) expressed in SFT (A) or DTF (B) in the analysis of the 3SEQ data. The criteria for inclusion on this list was the gene must show at least 100 reads across the DTF or SFT samples and be expressed exclusively in DTF or SFT or show at least 100 fold increased expression in DTF or SFT. (0.07 MB DOC) [file pone.0008768.s007.doc]

Table S5. Genes expressed exclusively (or almost exclusively) in DTF or SFT.

| A. Genes exclusively expressed in SFT: | |
| --- | --- |
| UCSC.Gene.ID | Gene Symbol |
| uc002coh.1 | NPW |
| AB058691 | AB058691 |
| uc001qaq.1 | NRGN |
| uc003xbj.1 | PHYHIP |
| uc001gxs.1 | PTPRV |
| uc001myb.1 | ALX4 |
| uc001fql.1 | C1orf92 |
| uc003ipl.1 | GRIA2 |
| uc002ltf.1 | LOC126520 |
| uc002ybr.1 | AK025855 |
| uc001llz.1 | KNDC1 |
| uc002wpm.1 | PCSK2 |
| uc003oqk.1 | DQ141194 |
| uc003wuc.1 | GATA4 |
| uc004epc.1 | CAPN6 |
| uc002zmp.1 | SLC25A18 |
| uc001ail.1 | LOC339457 |
| uc002ieq.1 | PYY |
| uc001dth.1 | GPR88 |
| uc003kls.1 | PCSK1 |
| uc001phj.1 | MMP3 |
| uc001has.1 | GOLT1A |
| uc001hhj.1 | HSD11B1 |
| uc001obs.1 | CDC42BPG |
| uc001hcf.1 | KLHDC8A |
| uc001cbv.1 | EPHA10 |
| uc001ijx.1 | FLJ45983 |
| uc003ioj.1 | FGG |
| uc002swt.1 | LOC90342 |
| uc002utf.1 | BC038548 |
| uc003paz.1 | GSTA1 |
| BC045182 | BC045182 |
| AK095503 | AK095503 |
| uc001oai.1 | SLC22A11 |
| uc001boc.1 | CD164L2 |
| AK310947 | AK310947 |
| DQ926657 | DQ926657 |
| A. Genes exclusively expressed in SFT (continued): | |
| UCSC.Gene.ID | Gene Symbol |
| AF086092 | AF086092 |
| AK095156 | AK095156 |
| X76978 | X76978 |
| uc001ldb.1 | VAX1 |
| AK094268 | AK094268 |
| uc001nry.1 | BC040894 |
| uc002lxr.1 | AF161441 |

| B. Genes exclusively expressed in DTF. | |
| --- | --- |
| UCSC.Gene.ID | Gene Symbol |
| AK095500 | AK095500 |
| uc002pua.1 | KLK4 |
| AK131543 | AK131543 |
| uc002fef.1 | CHST6 |
| uc002yek.1 | C20orf58 |
| uc003mjv.1 | CR598488 |
| uc001gwn.1 | TNNI1 |
| uc002ebx.1 | COX6A2 |
| uc001wjx.1 | MYH7 |
| uc003anz.1 | MB |
| uc002qjb.1 | TNNT1 |
| uc003ftt.1 | LRRC15 |
| uc002bmz.1 | ACAN |
| uc001ypv.1 | DKFZp686J02145 |
| uc003ylk.1 | CTHRC1 |
| uc003inv.1 | SFRP2 |
| uc001uwo.1 | POSTN |
| uc002rdo.1 | SDC1 |
